# The complete chloroplast genome sequence of *Melothria scabra* (Cucurbitaceae)

**DOI:** 10.1080/23802359.2024.2435901

**Published:** 2024-12-03

**Authors:** Chan Deng, Xinbi Jia, Siyue Chen, Jiaqi Guo, Chenghong Zeng, Yuewen Chen, Qianglong Zhu, Yingjin Huang

**Affiliations:** a Jiangxi Key Laboratory of Crop Physiology, Ecology and Genetic Breeding, Jiangxi Agricultural University, Nanchang, China; b Jiangxi Province Key Laboratory of Vegetable Cultivation and Utilization, Jiangxi Agricultural University, Nanchang, China

**Keywords:** Cucurbitaceae, chloroplast genome, *Melothria scabra*, phylogenetic analysis

## Abstract

*Melothria scabra* has gradually become an economically important plant worldwide. The complete chloroplast genome of *M. scabra* has a length of 156,744 bp, contains a large single-copy (LSC) region (86,387 bp), a small single-copy (SSC) region (18,055 bp), and two inverted repeats (IRs) with the same length of 26,151 bp. In total, 126 genes were detected, including 83 protein-encoding genes, 35 transfer RNA (tRNA) genes, and eight ribosomal RNA (rRNA) genes. For phylogenetic analysis, *M. scabra* has a closer genetic relationship with *Cucumis sativus* and *Citrullus lanatus*. The complete chloroplast genome sequence of *M. scabra* would promote the germplasm exploration, phylogenetic relationships, and molecular biology researches in *Melothria*.

## Introduction

*Melothria scabra* is commonly referred to as the cucamelon, penguins, mini watermelon, or sour cucumber (Chomicki et al. 2020). *M. scabra* is a diploid (2n = 2x = 24) species that originated in Mexico and Central America; now, it has successfully spread to Asia, Europe, and Africa (Bhowmick & Jha 2022). *M. scabra* has angular grooves on its stem and branches, bright green leaves, palmate serrated leaves, small and pointed melon leaves, yellow flowers, and thin vines. It grows rapidly, has a strong covering ability, and is often used as an ornamental plant in gardens (Tao et al. 2013; Kamaruddin et al. 2021).

The appearance of *M. scabra* is similar to that of a mini watermelon, but its flavor is similar to that of a cucumber (Tao et al. 2013). The fruit flesh is rich in vitamin C, lycopene, calcium, iron, and several other essential nutrients, making it a new popular fruit (Dunaevskaya & Kravchenko 2020). Furthermore, the ethanol, methanol, and ethyl acetate fractions of *M. scabra* were shown to exhibit significant antioxidant activity, indicating that *M. scabra* could be a promising source of natural antioxidant agents (Kamaruddin et al. 2021). A preliminary investigation demonstrated that *M. scabra* could have antidiabetic and hypoglycemic properties (Govindula et al. 2019). In addition, relatively high concentrations of the amino acids arginine and citrulline, which have anticancer properties, were detected in *M. scabra* (Roberts et al. 2018).

However, *M. scabra* shares some interspecific morphological traits with other related species in the Cucurbitaceae family, such as watermelon and cucumber, which leads to a perplexing taxonomy for *M. scabra*. Furthermore, the lack of genetic diversity analyses of *M. scabra* is hindering the effective development and utilization of germplasm resources. The complete chloroplast genome is widely used in plant species taxonomy, phylogenetic, and genetic diversity analyses (Song et al. 2022; Zhang et al. 2022; Liu et al. 2024). Compared with nuclear genomes, the chloroplast genome is mostly maternally inherited, with independent evolutionary routes, moderate evolutionary rates, low nucleotide substitution rates, and stable gene structures, which can easily provide genetic information for plant species taxonomy studies (Kaundun & Matsumoto 2011). Therefore, we sequenced, assembled, and annotated the chloroplast genome of *M. scabra* for the first time in this study.

## Materials and methods

### Plant material, DNA extraction, and sequencing

**Figure 1.**
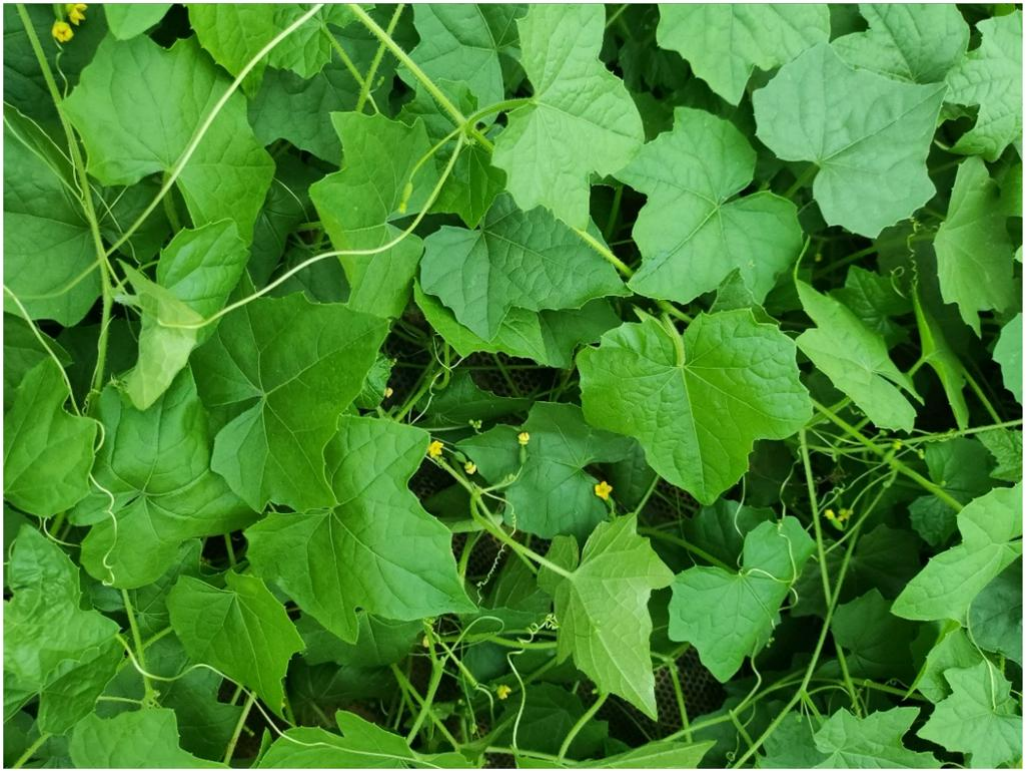
The growth picture of *M. scabra* (3520 × 2640). The image was taken in june 2024 by Chan Deng in the horticulture teaching practice base, at Jiangxi Agricultural University, Nanchang, China (N28°45′51″, E115°49′58″). *M. scabra* have bright green and small leaves, curly vines, and yellow flowers. Meanwhile, the fruit is soval with an average length of 2.4 cm and a width of 1.5 cm.

*M. scabra* (voucher number: QZ01MS, Figure 1) seeds were collected from a cultivated population in October 2023 in Feidong County (N31°93′12.81″, E117°63′96.69″), Anhui Province, China. They were subsequently planted in the horticulture teaching practice base at Jiangxi Agricultural University, Nanchang, China (contact person: Chan Deng, dengchan2023@163.com). During the healthy growth of plants, healthy and fresh leaves were selected in November 2023, and their total genomic DNA was extracted *via* a modified cetyltrimethylammonium bromide (CTAB) method (Li et al. 2013). The concentration and quality of the DNA samples were detected *via* agarose gel electrophoresis and a Nanodrop-2000 nucleic acid assay, and the DNA samples that met the requirements were used for subsequent experiments. Subsequently, 20 μg of isolated gDNA was sent to Bena Technology Co. Ltd. for genome sequencing *via* the Illumina NovaSeq 6000 platform (Wuhan, China).

### Chloroplast genome assembly and annotation

The raw pair-end (PE) reads were filtered for quality control to ensure the high quality of the clean PE reads *via* NGSQCToolkit v2.3.3. The high-quality clean PE reads were randomly selected *via* Seqtk, and then the clean PE reads were assembled into contig sequences *via* the Plasmidspades.py script program in SPAdes v3.6.1 (Bankevich et al. 2012). The scaffolds were subsequently aligned to the chloroplast reference genome of *Citrullus lanatus* (NC_032008.1) *via* BLASTN (Talamantes et al. 2021), and the draft chloroplast genome of *M. scabra* was constructed.

The gaps in the draft chloroplast genome of *M. scabra* were filled *via* GapCloser. The complete chloroplast genome sequence was subsequently annotated *via* CPGAVAS2 and GeSeq (Tillich et al. 2017; Shi et al. 2019), and the annotation results were subsequently checked and corrected *via* Sequin. A circular map of the complete chloroplast genome of *M. scabra* was constructed *via* CPGView (Liu et al. 2023).

### Phylogenetic analysis

To clarify the phylogenetic relationships among *M. scabra* and other related species in Cucurbitaceae in this study, the complete chloroplast genome sequences were aligned *via* MAFFT, v7.463 (Rozewicki et al. 2019). The phylogenetic tree was constructed *via* MEGA v11 (Tamura et al. 2021) with the maximum likelihood (ML) method and 1000 bootstrap values.

## Results

### General features of the chloroplast genome

**Figure 2.**
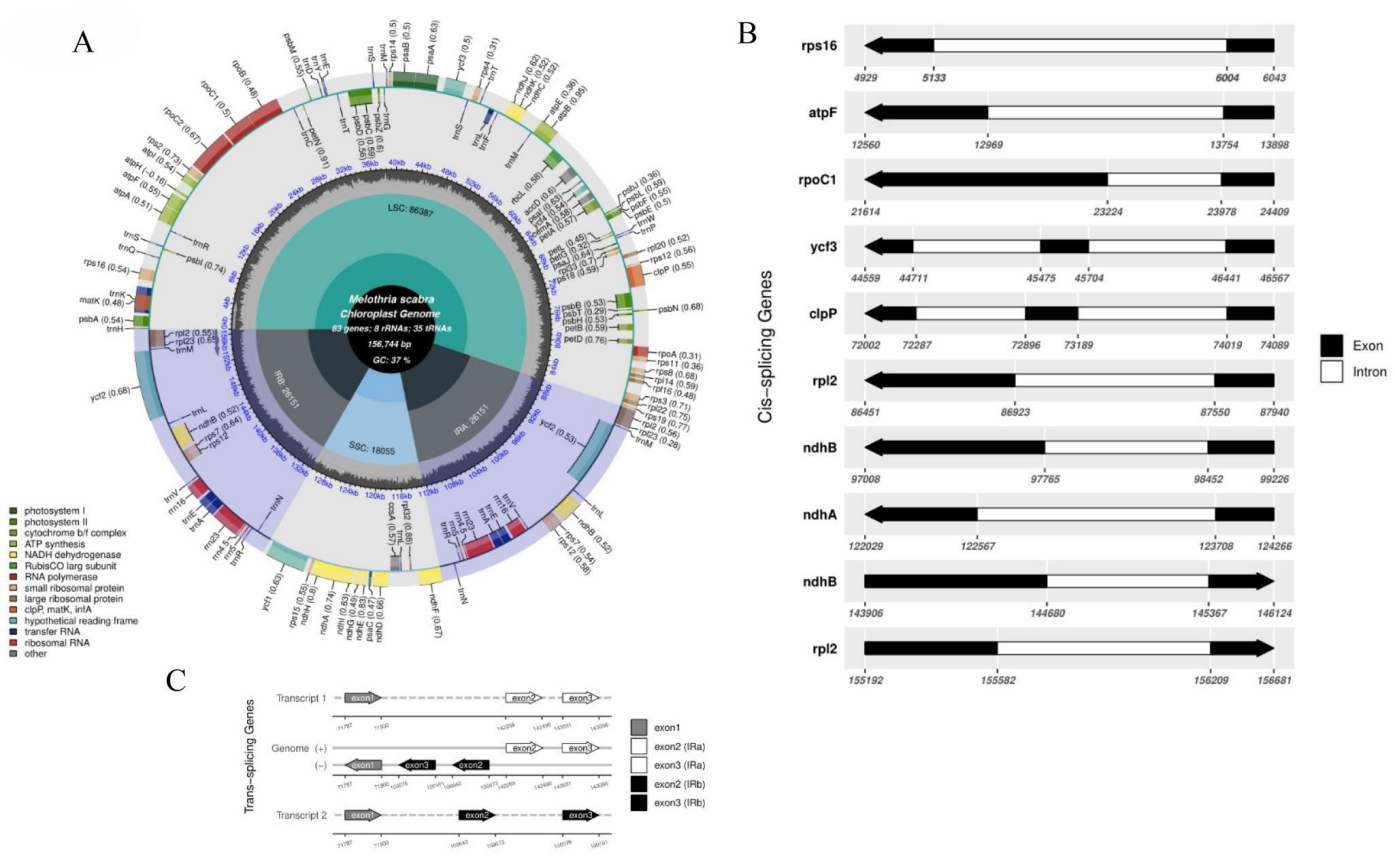
The complete chloroplast genome map of *M. scabra*. (A) The genes are arranged on the circular chloroplast genome with different colored boxes representing specific gene regions. The genes transcribed in clockwise and counterclockwise directions are shown on the inner and outer sides of the circle, respectively. Additionally, the GC content is presented *via* the grey area in the center of the circle, while the four-part structures of LSC, SSC, IRa, and IRb are depicted on the inner circle. (B) The black-white arrow showed the cis-spliced genes, and (C) The black-grey-white arrow showed trans-spliced genes.

A 0.4 Gb of high-quality clean PE reads was extracted from total sequence data for chloroplast genome assembly. The complete chloroplast genome of *M. scabra* is 156,744 bp in length and has a typical quadripartite structure (Figure 2A). The assembled complete chloroplast genome presented an average read mapping depth of 4174.60 × and a minimum mapping depth of 67 × (Figure S1). It contains LSC (86,387 bp) and SSC (18,055 bp) regions and two IRs (26,151 bp), and its total GC content is 37.19%. Furthermore, a total of 126 genes were annotated, including 83 protein-coding genes, 35 tRNA genes and eight rRNA genes. Among these genes, eleven (*ndhB, rpl23, rps7, rrn16S, rrn4.5S, rrn5S, trnE-UUC, trnL-CAA, trnN-GUU, trnV-GAC, ycf2*) are duplicates, six protein-coding genes (*rps16, atpF, rpoC1, rpl2, ndhB, ndhA*) have a single intron, and two protein-coding genes (*ycf3, clpP*) have two introns (Figure 2B and 2C). The annotated complete chloroplast genome of *M. scabra* was submitted to the sequence database of CNGBdb with the accession number N_001486263.

### Phylogenetic relationship analysis

**Figure 3.**
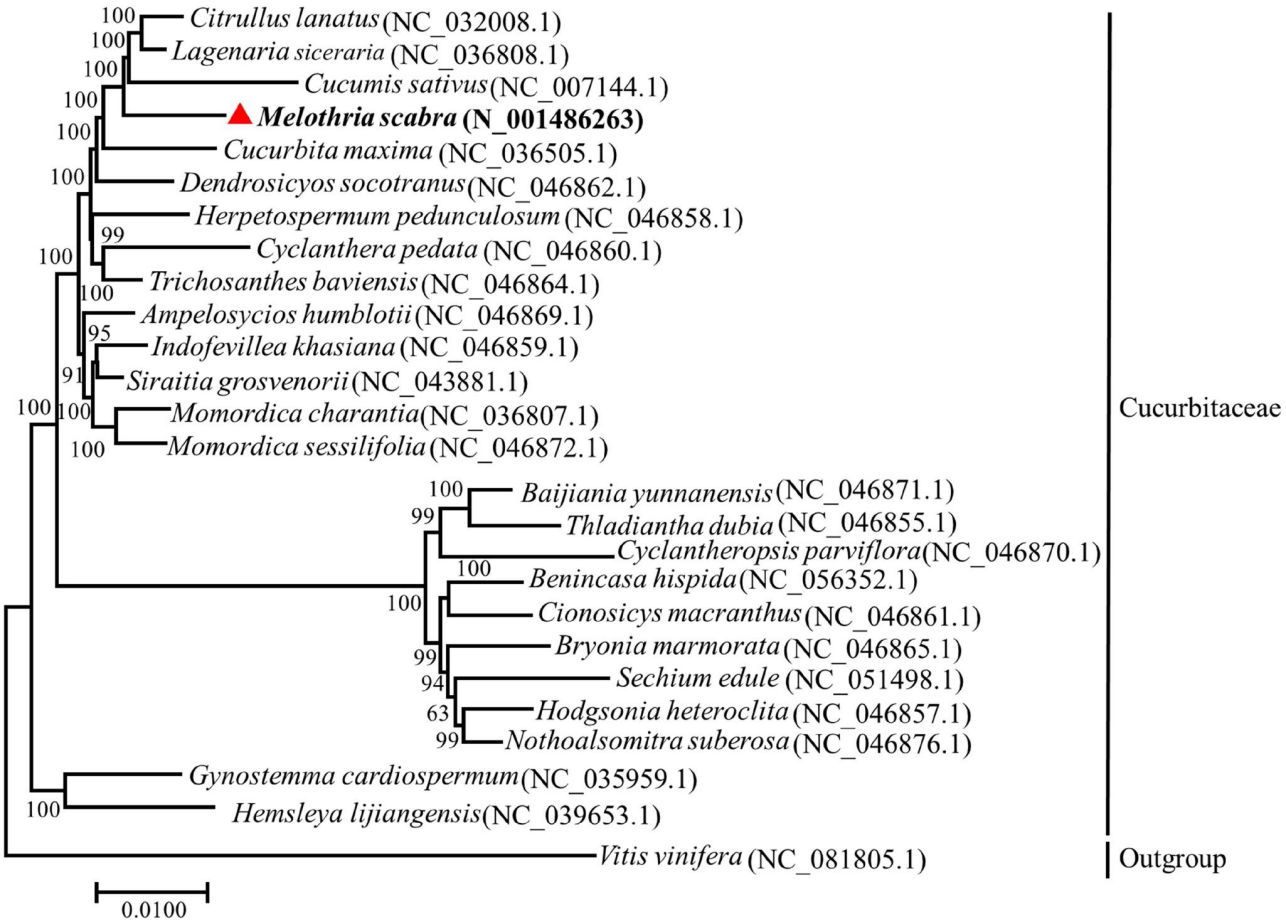
The phylogenetic tree suggested the relationship between *M. scabra* and 25 other species within the family Cucurbitaceae. The *Vitis vinifera* L. was taken as an outgroup. The maximum-likelihood method was used to infer the position of the *M. scabra* in the phylogenetic tree, using 1000 as the bootstrap value and the number of upper branches as the bootstrap value. The following GenBank sequences were used: *Ampelosycios Hublot* NC_046869.1, *Bryonia marmorata* NC_046865.1, *Baijiania yunnanensis* NC_046871.1, *Benincasa hispida* NC_056352.1, *Citrullus lanatus* NC_032008.1 (Zhu et al. 2016), *Cucumis sativus* NC_007144.1 (Plader et al. 2007), *Cucurbita maxima* NC_036505.1, *Cyclanthera pedata* NC_046860.1, *Cyclantheropsis parviflora* NC_046870.1, *Cionosicys macranthus* NC_046861.1, *dendrosicyos socotranus* NC_046862.1 (Bellot et al. 2020), Gynostemma Cardiospermum NC_035959.1, *Herpetospermum pedunculosum* NC_046858.1, *Hodgsonia heteroclita* NC_046857.1, *Hemsleya lijiangensis* NC_039653.1 (Zhang et al. 2018), *Indofevillea khasiana* NC_046859.1, *Lagenaria siceraria* NC_036808.1, *Momordica sessilifolia* NC_046872.1, *Momordica charantia* NC_036807.1, *Nothoalsomitra suberosa* NC_046876.1, *Siraitia grosvenorii* NC_043881, *Sicyos edule* NC_051498.1 (Cui et al. 2021), *Trichosanthes baviensis* NC_046864.1, *Thladiantha dubia* NC_046855.1, *Vitis vinifera* NC_081805.1 (Wang et al. 2021).

To date, the complete chloroplast genome of *M. scabra* is the first to be published in the genus *Melothria*, with 13 species. More than 60 complete chloroplast genomes belonging to 24 genera in the family Cucurbitaceae have been published in the NCBI genome database. To confirm the phylogenetic status of *M. scabra*, 25 complete chloroplast genome sequences representing the different genera of Cucurbitaceae and an outgroup species (*Vitis vinifera*) were used to infer a phylogenetic tree. A phylogenetic tree was constructed to explore the phylogenetic relationships between *M. scabra* and other Cucurbitaceae plants. The bootstrap value for the clade of *M. scabra* with other close relatives was 100, indicating that the phylogenetic tree has high reliability. The inferred phylogenetic tree revealed that *M. scabra* fell between Cucurbita and Cucumis, suggesting that *M. scabra* is a member of the family Cucurbitaceae. Moreover, *M. scabra* is closely related to *Cucumis sativus* and *Citrullus lanatus*, confirming that *M. scabra* shares interspecific morphological traits with *Cucumis sativus* and *Citrullus lanatus* (Figure 3).

## Discussion and conclusion

In this study, the complete chloroplast genome of *M. scabra* was sequenced, assembled de novo, annotated, and phylogenetic analysis was conducted with another related species in the Cucurbitaceae family. The chloroplast genome of *M. scabra* is 156,744 bp in size with a typical circular tetrameric structure and contains LSC and SSC regions and two IR regions. All 126 genes were identified, including 83 protein-encoding genes, 35 tRNA genes, and eight rRNA genes. There was no marked difference in the chloroplast genome structure or gene content between *M. scabra* and other related species in the Cucurbitaceae (Li et al. 2022; Zhou et al. 2023). Moreover, phylogenetic relationship analysis revealed that *M. scabra* belongs to the family Cucurbitaceae, which confirmed the phylogenetic relationships of *M. scabra* proposed in earlier studies (Kocyan et al. 2007; Chomicki et al. 2020). Furthermore, *M. scabra* and three other species (*Cucumis sativus*, *Lagenaria siceraria*, and *Citrullus lanatus*) were grouped into a clade, suggesting that the four species have relatively close genetic distances and that *M. scabra* could be the closest wild ancestor of the other three species. This study also supported the early conclusion that *M. scabra* looks like a mini watermelon and has a cucumber-like flavor (Tao et al. 2013). Our research results could be used to analyze the genetic diversity and phylogenetic relationships of *M. scabra* and its related species within the family Cucurbitaceae.

## Supplementary Material

supplementary material.docx

## Data Availability

The data supporting the findings of this study is available in CNGBdb (https://db.cngb.org) with the accession number: N_001486263. The associated project, sample, and experiment numbers are CNP0005296, CNS1008401, and CNX0953946 in the CNGBdb database, respectively.
