# Supplementary figures and images for: The complete chloroplast genome sequence of Melothria scabra (Cucurbitaceae)
Source: Mitochondrial DNA B Resour. 2024 Dec 3;9(12):1648–52. doi: 10.1080/23802359.2024.2435901 (PMC11619021; doi:10.1080/23802359.2024.2435901)

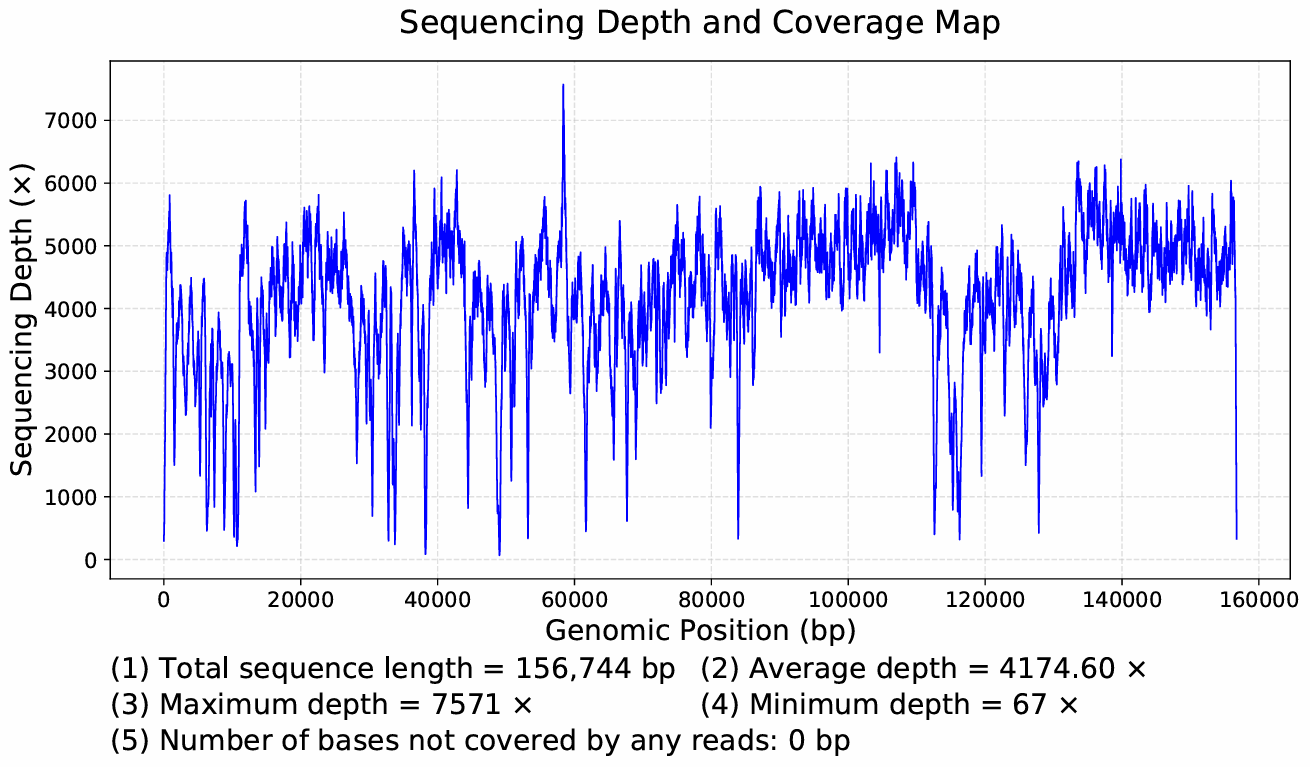


Figures S1. 3177x2308mm (600 x 600 DPI)

Supplement: supplementary material.docx [file TMDN_A_2435901_SM4903.docx]
